# Preliminary study on HIV status disclosure to perinatal infected children: retrospective analysis of administrative records from a pediatric HIV clinic in the southern United States

**DOI:** 10.1186/s13104-020-05097-z

**Published:** 2020-05-24

**Authors:** Henna Budhwani, Lori Mills, Lauren E. B. Marefka, Sequoya Eady, Van T. Nghiem, Tina Simpson

**Affiliations:** 1 Department of Health Care Organization and Policy, School of Public Health, University of Alabama at Birmingham (UAB), Birmingham, AL 35294 USA; 2 Department of Pediatrics, School of Medicine, University of Alabama at Birmingham (UAB), Birmingham, AL 35294 USA

**Keywords:** HIV, Disclosure, Viral load, Adolescents, Children, People living with HIV

## Abstract

**Objective:**

The World Health Organization recommends disclosing HIV-status between 6 and 12 years; American Academy of Pediatrics recommends that children are informed at “school age.” Neither suggests an optimal age when children should learn of their status to improve viral load suppression. Considering that virally suppressed people do not transmit HIV and that interrupting the transmission cycle is critical to ending the HIV epidemic, our objective is to examine the relationship between age of disclosure and viral load suppression by evaluating data from a pediatric HIV clinic in the southern United States. Records from perinatal infected patients seen between 2008 and 2018 were analyzed (N = 61).

**Results:**

Longitudinal suppression was low across all groups when benchmarked against the UNAIDS 90% global target; black patients were less likely to achieve suppression compared to white patients (41% vs. 75%, *p *= 0.04). Adopted children were more likely to achieve suppression than children living with biological family (71% vs. 44%, *p *< 0.05). Children who learned of their status between 10 and 12 had the highest rate of suppression (65%) compared to peers who learned of their status younger (56%) or older (38%). Our preliminary study is designed to spark research on refining the current recommendations on HIV-status disclosure to perinatal infected children.

## Introduction

There are 1.8 million children (< 15 years of age) living with human immunodeficiency virus (HIV) globally—many of these children were infected perinatally, and nearly 2000 of these children living with HIV reside in the United States [1]. Addressing suboptimal rates of viral load suppression in the perinatal infected population is an urgent clinical and public health priority [2, 3]. When an infant is born with HIV, a guardian must inform the child that they are living with HIV and explain what that means. This discussion may include topics on the infectious nature of HIV, that the child contracted HIV from the biological mother, and the need for the child to stay adherent to antiretroviral medications to ensure continued viral suppression—all of which require the child or youth living with HIV (YLWH) to intellectually comprehend the content of these discussions, and hopefully, to not internalize HIV-related stigma or act out against a guardian who obfuscated a diagnosis for years. Considering the importance of knowing one’s HIV-status, the World Health Organization recommends disclosing HIV-status to perinatal infected children between 6 and 12 years; American Academy of Pediatrics recommends YLWH are informed at “school age,” but neither organization suggests an optimal age when YLWH should learn of their HIV-status to promote well-being [4, 5]. Considering the importance of viral suppression to stopping HIV transmission, the objective of this retrospective, preliminary study of administrative records is to explore if there is potential to narrow the recommended age range at which perinatal infected YLWH should learn of their HIV status to promote long-term viral suppression.

## Main text

### Data and study participants

We examined records of perinatal infected YLWH who received care over a 10-year period (2008–2018) at a pediatric HIV clinic in the southern United States (n = 61). Although records were extracted from the clinic’s electronic medical records database, older records existed as scanned files within the system, requiring hand extraction of study data. Behaviorally-infected patients were excluded. At the time these data were extracted (2019), the age range of study participants was 14 to 32 years; however, records were only evaluated for the time during which these individuals were active patients at our study site aged 0 to 24 years (historically, YLWH aged out of HIV pediatric care at 25). Because this study is a retrospective analysis of administrative data, there were no control conditions, and there was no study population monitoring process. Perinatal infected YLWH treated at this clinic over the defined 10-year period, who were aware of their HIV status, were included in this study. Patients were excluded from this analysis under two conditions: patient was unaware of HIV status (not yet disclosed) and/or if the patient did not have a Ryan White consent form on record.

### Measure of interest, age of disclosure

Age of HIV-status disclosure was extracted from administrative clinical records as a continuous variable. Age of HIV-status disclosure was collected through self-report from the patient or the patient’s guardian typically in the presence of the patient. We collapsed age at which YLWH learned of their status into three categories based on the developmental stages of children and adolescents: learned of HIV-status younger than 10 years (children), between 10 and 12 (pre-adolescents), and those 13 and older (adolescents). For patients who were unaware of their HIV-status at the time of this analysis, age of disclosure was marked as “missing.”

### Measure of interest, viral load

Viral load is a key indicator along the HIV continuum of care and was assessed by quantitative HIV ribonucleic acid (RNA) polymerase chain reaction (PCR) that was conducted through laboratory assays. For YLWH, viral loads may be assessed multiple times a year. We evaluated the annual viral load that was closest to the patient’s birthday, beginning at 14 years and concluding at 18 years (one viral load annually). Viral suppression was indicated when the plasma HIV RNA laboratory result was under 200 copies/mL, as recommended by the Health Resources and Services Administration [6]. This suppressed result indicated that the assay was unable to detect HIV RNA within the patient’s plasma specimen. For each annual numeric viral load, we constructed an associated, binary measure to indicate if the patient was suppressed or not suppressed. These measures were populated as 1 = suppressed if laboratory results indicated viral load < 200 (HIV RNA PCR), and for viral loads equal to or above 200, 0 = unsuppressed. Missing viral loads were coded as 9 = missing. This data management process produced five independent categorical variables indicating if the patient was suppressed at five sequential time points.

We then used these annual categorical measures of viral suppression to construct a single binary measure of multi-year longitudinal viral suppression. If 80% or more of the annual viral loads were 1 = suppressed, then the longitudinal viral load measure was coded as 1 = yes. If a patient had less than an 80% suppression rate, the measure was coded 0 = no, for not longitudinally suppressed. For example, if records indicated that the patient’s viral loads were suppressed across four of five annual laboratory results (80%), then this constructed measure was coded as 1 = suppressed. We identified 80% as our threshold, because—assuming the maximum of five viral loads on record—this allowed patients to be classified as longitudinally suppressed with one unsuppressed annual viral load on file.

### Statistical assessment and ethical approval

We conducted Chi squared tests for comparisons of characteristics for proportions. All the tests were two-sided (α < 0.05) and conducted in SAS Enterprise Guide 8.1 (SAS^®^ Institute Inc.). University of Alabama at Birmingham (UAB) provided ethical approval (IRB-981112002).

### Results

#### Sample characteristics

Average length of time that YLWH were unaware of their status was about 10 years. Nearly three-quarters (73%) of study participants were diagnosed with HIV prior to their first birthday. Eighty percent of our sample identified as non-White (Black, Hispanic, and Asian); two-thirds (66%) identified as Black-only or African American; 49% were male (51% female; none identified as transgender). About 40% were adopted and 60% lived with a biological family member. Of the 40% of adoptees, 57% were domestic adoptions, and 43% were adopted from international settings. Of the 60% living with biological family, 63% were living with a biological parent, while 39% were living with extended family.

#### Viral suppression

We present the results of our analyses in Table 1. Longitudinal suppression was low across all groups when benchmarked against the UNAIDS 90% global target [7]. See Table 1. Of the fifty-nine patients with at least one viral load on record, 51% were longitudinally suppressed; 30% of black YLWH compared to 75% of white YLWH were longitudinally suppressed. Overall, black YLWH were less likely to achieve 80% + longitudinal suppression compared with the rest of the group (41% vs. 71%, *p *= 0.02) or compared with white YLWH (41% vs. 75%, *p *= 0.04). Internationally-adopted children had a greater than 90% rate of longitudinal suppression. Domestically adopted YLWH, YLWH living with extended family, and YLWH living with a biological parent had suppression rates ranging between 42 and 46% (46%, 42%, and 43% respectively). Among patients with known age of HIV-status disclosure (n = 53), adopted children were statistically more likely to achieve 80% + longitudinal suppression compared to children living with biological parent(s) or extended family members (71% vs. 44%, *p *< 0.05). Children who learned that they were living with HIV prior to age 10 were longitudinally suppressed at a rate of 56%. The rate of longitudinal suppression climbed to a notable 65% in YLWH who learned of their status between 10 and 12. The lowest rate of suppression (38%) was in adolescents who learned of their HIV-status at 13 or older. Although substantively important, we did not find that these differences were statistically significant (likely due to small sample size).

**Table 1:** Characteristics of those who learned of their HIV-status across three age bands

Age of HIV-status disclosure	80% + suppression%, (n =)	Black only%, (n =)	Adopted%, (n =)
Under 10 (n = 9)	56% (5)	56% (5)	44% (4)
10–12 years (n = 26)	65% (17)	62% (16)	46% (12)
13 or older (n = 19)	44% (8)	68% (13)	26% (5)

### Discussion

Our preliminary findings suggest that disclosing HIV-status between 10 and 12 may promote viral suppression through medication adherence. This period of pre-adolescence, between 10 and 12, is when youth have developed greater intellectual capacity and emotional resilience compared to younger peers. Youth at this age are more likely to comprehend medical explanations and healthcare considerations related to living with HIV and how HIV-status may influence social relationships compared to younger peers; youth in this age range are potentially less likely, due to lower levels of autonomy and independence, to rebel against caregivers who have obfuscated their HIV-status for years [8, 9]. Considering the potential relationship between living arrangement and viral suppression, we reviewed our data to see if adopted children were over-represented in the 10 to 12 age group. We did not find that these rates differed in a substantive way between those who learned of their status under the age of 10 and those who learned of their status between 10 and 12 years.

## Limitations

Limitations should be considered when extending our findings. Small sample size restricted our ability to conduct advanced statistical analyses. Further research analyzing pooled multi-clinic data can replicate, refine, and validate findings. Age of disclosure was self-reported. Although self-reported outcomes are subject to recall bias, the process of disclosing HIV-status is often supported by clinical providers; thus, age of disclosure is captured quickly after the disclosure occurs.

## Data Availability

The data for this study may be made available through request to the senior author.

## Abbreviations

HIV: Human immunodeficiency virus
PCR: Polymerase chain reaction
RNA: Ribonucleic acid
YLWH: Youth living with HIV
